# Change in skeletal muscle associated with unplanned hospital admissions in adult patients: A systematic review and meta-analysis

**DOI:** 10.1371/journal.pone.0210186

**Published:** 2019-01-04

**Authors:** Peter Hartley, Patricia Costello, Rachel Fenner, Nathalie Gibbins, Édáin Quinn, Isla Kuhn, Victoria L. Keevil, Roman Romero-Ortuno

**Affiliations:** 1 Department of Public Health and Primary Care, University of Cambridge, Cambridge, United Kingdom; 2 Department of Physiotherapy, Cambridge University Hospital NHS Foundation Trust, Cambridge, United Kingdom; 3 Medical Library, University of Cambridge, Cambridge, United Kingdom; 4 Department of Medicine for the Elderly, Cambridge University Hospital NHS Foundation Trust, Cambridge, United Kingdom; 5 Discipline of Medical Gerontology, Trinity College Dublin, Mercer’s Institute for Successful Ageing, St James’s Hospital, Dublin, Ireland; Edge Hill University, UNITED KINGDOM

## Abstract

**Objectives:**

The primary objective of the review was to describe change that occurs in skeletal muscle during periods of unplanned hospitalisation in adult patients. The secondary objective was to examine the relationship between both physical activity and inflammation with the change in skeletal muscle. A further objective was to investigate the effect of interventions on change in skeletal muscle during periods of unplanned hospitalisation.

**Design:**

A systematic review and meta-analyses. Embase, MEDLINE, CINAHL, AMED, PEDro and the Cochrane Library were searched for studies that included any measures of skeletal muscle (excluding pulmonary function) at two time points during unplanned hospitalisation. Studies that were set in critical care, or included patients with acute or progressive neurological illness, were excluded.

**Results:**

Our search returned 27,809 unique articles, of which 35 met the inclusion criteria. Meta-analyses of change between baseline and follow-up in random effects models suggested that grip strength had an average increase: standardised mean difference (SMD) = 0.10 (95% CI: 0.03; 0.16); knee extension strength had an average reduction: SMD = -0.24 (95% CI: -0.33; -0.14); and mid-arm muscle circumference had an average reduction: SMD = -0.17 (95% CI: -0.22; -0.11). Inflammation appeared to be associated with greater loss of muscle strength. There was inconclusive evidence that the level of physical activity affects change in skeletal muscle. In regard to the effect of interventions, only exercise interventions were consistently associated with improved skeletal muscle outcomes.

**Conclusions:**

Adult patients who undergo an unplanned hospital admission may experience a small reduction in knee extension strength and mid-arm muscle mass. Prospective research is needed to clarify the contribution of confounding factors underlying the observations made in this review, with particular attention to levels of physical activity, and possible contributions from environmental factors and processes of hospital care.

## Introduction

In older patients, it is estimated that the risk of having a reduction in independence to carry out activities of daily living during a hospital admission is around 30% [1]. Although the majority of literature focuses on older adults, there is evidence that adults below the age of 60 are at high risk of functional decline as well [2]. Terms such as hospital-associated functional decline [3] and hospital-associated deconditioning [4] have been used to refer to that loss of functional ability. Whilst mechanisms are not well understood, one is thought to be skeletal muscle wasting and/or loss of muscle strength [5, 6], through disuse as a consequence of bed rest. However, reduced nutritional intake and inflammation as a result of acute illness are also thought to contribute [5–7]. In addition, some patients may be more vulnerable to functional loss whilst hospitalised [1, 8], and the hospital processes of care including environmental factors may also play a role [3].

A review has highlighted the abundant evidence of the negative effect of bed rest on skeletal muscle in healthy volunteers both young and old [9]. These changes include loss of strength, mass and protein synthesis [9]. Although it is established that physical activity in most patients during periods of hospitalisation is low [10] [11, 12], it is unclear whether studies of complete bed rest in healthy volunteers can be extrapolated to hospitalised individuals.

The primary objective of this review was to describe change that occurs in skeletal muscle during periods of unplanned hospitalisation in adult patients. The secondary objective was to examine the relationship between both physical activity and inflammation with the change in skeletal muscle during periods of hospitalisation. A further objective was to investigate the effect of interventions on change in skeletal muscle during periods of unplanned hospitalisation in adult patients.

## Method

### Search strategy

A protocol for this review was registered on PROSPERO: CRD42016046590 (https://www.crd.york.ac.uk/prospero). The following databases were searched electronically between inception and October 2017: Embase via OVID, MEDLINE via OVID, CINAHL via EbscoHOST, AMED via OVID, PEDro and the Cochrane Library. The MEDLINE search strategy is presented in the supplementary material (S1 Text). If a conference abstract or dissertation was identified that appeared relevant, searches were made to identify a full paper; if no paper was found the authors of the abstract were contacted. The reference lists of included studies, identified reviews and our own personal literature databases were searched to identify any potential studies additional to those identified through the electronic searching. Only English language articles were eligible for inclusion.

### Selection criteria

#### Population

Adult patients (aged ≥18 years) who experience an unplanned (i.e. non-elective) admission to hospital. Studies that focused on patients with an acute or progressive neurological condition were excluded.

#### Setting

Acute hospital ward (i.e. excluding sub-acute or intermediate care such as inpatient rehabilitation) and excluding critical care units.

#### Outcomes

Any measure of skeletal muscle excluding measures of pulmonary function/spirometry. For the study to be included, at least 2 measures of skeletal muscle had to be taken whilst the patient was in hospital; the first measure within 72 hours of hospital admission (T0), and the second at least a day later (T1). The skeletal muscle was not to be affected by acute trauma (including surgical).

### Study selection

Reviewers worked independently using the pre-set inclusion criteria to identify relevant studies. The reviewers screened the articles’ titles and abstracts and classified each as relevant, not relevant or unsure. All articles screened by two reviewers as being not relevant were excluded. The reviewers then independently reviewed all other papers in full, but only using classifications of relevant or not relevant. Any discrepancy or uncertainty regarding the eligibility of a study was discussed between the two reviewers (who read the full paper together) or with a third author until consensus was reached. If variables of interest were measured but not reported, attempts were made to contact the authors before classifying a study as not relevant. Following this, all articles classified as not relevant were excluded from the review and the reasons were documented.

### Data extraction

Data was extracted from studies using a pre-designed table. If key data variables were missing, authors were contacted for unpublished data. Key variables included: average age, setting (acute versus sub-acute), T0 time, time between T0 and T1, average and measure of variance of outcomes of interest at T0, T1 and change between T0 and T1.

### Analysis

Meta-analysis was performed when 3 or more studies measured the same outcome and sufficient data was published or made available by the authors. If there were insufficient studies or data available, a narrative summary of the data was produced. All meta-analyses used a random effects model. Inter-study heterogeneity was assessed using the I^2^ statistic [13] and 95% prediction intervals (PI) [14]. With outcomes that were assessed using different measurement methods but it was considered appropriate to group together (e.g. muscle strength, measured as torque or force) the standardised mean difference (SMD) with Hedge’s g correction was calculated [15]. When describing the change in skeletal muscle during periods of hospitalisation, the SMD was calculated as the mean difference divided by the standard deviation for within groups [15]. When comparing an intervention and control group, the SMD was calculated as the mean difference (MD) divided by the standard deviation of change [15].

Where data needed for meta-analysis was not presented in the original paper, the authors of the paper were contacted to request the necessary data. If this data was not received the paper was not included in the meta-analysis, but included in the review in a narrative summary.

Where data imputation was necessary, we used the methodology described in the Cochrane handbook [16]. If the standard deviation of change was not reported, this was estimated using the average correlation coefficient of the studies with full data reported, using methods described by Higgins & Green [16]. If data were presented as median and interquartile range (IQR), median scores were substituted for the mean values, and standard deviation estimated as IQR/1.35.

Outcome data which was reported by studies in separate sample groups (e.g. male and female) were combined so as not to disproportionately weight one study within the meta-analysis [16].

Sub-analysis was performed by separating studies that used fixed time points for outcome data collection (e.g. T1 being day 7 of hospitalisation) and studies that used variable time points based on the participants’ length of hospital stay (e.g. T1 being day of discharge). This was decided upon as it was felt that studies using a variable time point were likely to include a more heterogeneous sample.

Where 10 or more studies were included in a meta-analysis, a meta-regression analysis was performed using a mixed-effects model to analyse the influence of age on the degree of heterogeneity seen in the results.

Some of the included manuscripts referred to the day of admission to hospital as day 0 and some as day 1. For the purposes of this paper, we refer to the day of admission to hospital as day 1 and the second day in hospital as day 2, and have summarised all studies accordingly. If it was unclear from the manuscript if the authors were referring to the day of admission as day 1 or day 0, then the authors were contacted to clarify, if no response was received we assumed that day 1 or ‘first day’ referred to the day of admission.

Meta-analysis was performed with R software (version 3.3.2) [17] using the metafor package [18].

### Risk of bias

Randomised control trials (RCTs) and interventional observational studies were assessed using the Downs and Black’s tool [19], and non-interventional observational studies were assessed using the National Institute of Health’s Quality Assessment Tool for Observational Cohort and Cross-Sectional Studies [20]. All assessments were caried out at study level.

Publication bias was assessed with visual inspection of funnel plots and the Egger regression test for funnel plot asymmetry when ≥ 10 studies were included in the meta-analysis.

## Results

**Fig 1:**
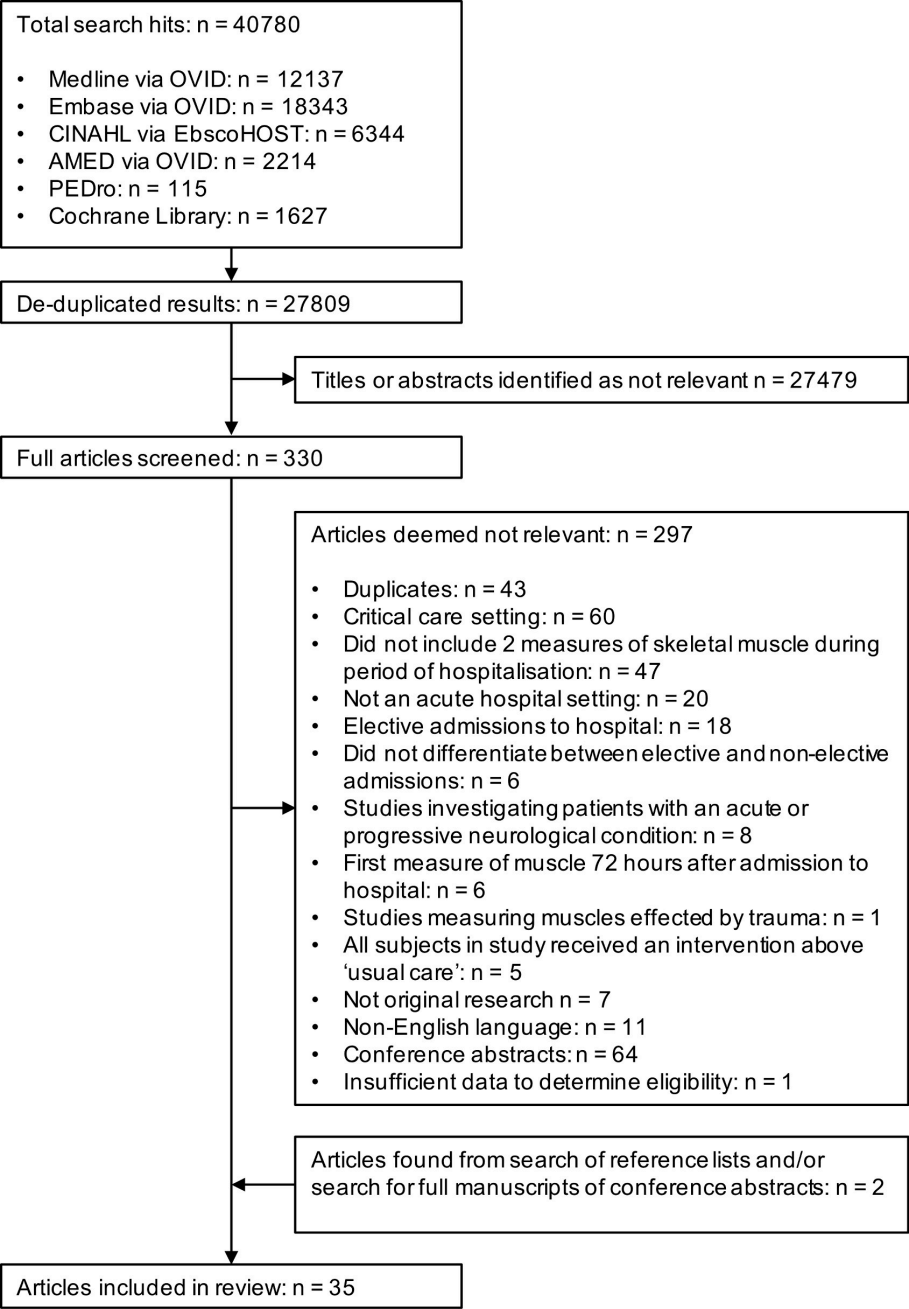
Summary of retrieval and review of articles.

Our search returned 27,809 unique articles, of which 35 met the inclusion criteria (Fig 1). Of the 35 studies, 18 were cohort studies [21–38], 11 were RCTs [39–49], and 6 were case control studies [50–55]. In total, there were 2489 hospitalised patients included at baseline assessment, of which 2264 received ‘usual care’. Studies are summarised in Table 1 and the critical appraisal results are summarised in S1 and S2 Table.

**Table 1:** Summary of included articles.

Study ID Country	Design	Key inclusion criteria	Time points outcome measures collected	Average age (mean ± SD)); % female	Outcomes of interest to this review
**Weinsier et al. 1979 [21]** **United States of America**	Cohort study	Age: NS Condition: NS Setting: General medical service	T0: Within 48 hours of admission (n = 144) T1: T0 + ≥ 2 weeks (based on LOS) (n = 44)	52 years; 50% female	1. Mid arm muscle circumference
**Abad et al. 1986 [22]** **Spain**	Retrospective cohort study	Age: NS Condition: Inflammatory bowel disease Setting: NS	T0: Admission (n = 47) T1: NS (n = 47)	36 years; % female NS	1. Mid arm muscle circumference
**Sloan et al. 1992 [39]** **Canada**	RCT CG: weekly placebo injection TG: weekly nandrolone injection	Age: ≥ 65 years Condition: Hip fracture Setting: Orthopaedic service	T0: Within 48 hours of admission (CG n = 14, TG n = 15) T1: T0 + 1 week (CG n = 14, TG n = 15) T2: T0 + 2 weeks (CG n = 14, TG n = 15) T3: T0 + 3 weeks (CG n = 9, TG n = 14) T4: T0 + 4 weeks (CG n = 7, TG n = 12)	CG: 81 ± 6 years; 100% female TG: 83 ± 7 years; 100% female	1. Mid arm muscle circumference 2. Lean body mass (bioelectrical impedance) 3. Handgrip strength
**Potter et al. 1995 [23]** **United Kingdom**	Cohort Study	Age: NS Condition: NS Setting: Geriatric Unit	T0: Admission (n = 69) T1: T0 + 2 weeks (n = 41) T2: T0 + 4 weeks (n = 20) T3: T0 + 6 weeks (n = 12)	82.2 (range: 69–96) years; 65.2% female	1. Mid arm muscle circumference 2. Handgrip strength
**Unosson et al. 1995 [24]** **Sweden**	Cohort Study	Age: ≥ 70 years Condition: Hip fracture Setting: Orthopaedic department	T1: Pre-operative T0: T0 + 2 weeks	84.6 years; 84% female	1. Mid arm muscle circumference 2. Lean body mass (bioelectrical impedance)
**Antonelli** **Incalzi et al.** **1996 [25]** **Italy**	Cohort study	Age: ≥ 70 years Condition: NS Setting: Medicine and Geriatric wards	T0: Within 24 hours of admission (n = 302) T1: Discharge (n = 283) LOS: 22.9 ±17.2	78.8 ± 5.8 years; 53.3% female	1. Mid arm muscle circumference
**Saudny-Unterberger et al. 1997 [40]** **Canada**	RCT CG: usual care TG: usual care + ‘aggressive oral nutritional support’;	Age: 40–85 years Condition: COPD Setting: Respiratory medicine service	T0: Admission (CG: n = 16, TG = 17) T1: T0 + 2 weeks (CG: n = 10, TG = 14)	CG: 69.4 ± 3.91 years; 30% female TG: 69.21 ± 2.21 years; 43% female	1. Handgrip strength
**Gupta et al. 2001 [26]** **United Kingdom**	Cohort study	Age: ≥ 16 years Condition: severe pancreatitis Setting: NS	T0: Day 0 (n = 17) T1: Day 3 (n = 17) T2: Day 7 (n = 17)	Median: 60 (range: 38–89) years; 59% female	1. Handgrip strength 2. Mid arm muscle circumference
**Humphreys et al. 2002 [50]** **Chile**	Case control study	Age: NS Condition: NS Setting: Medical and surgical wards	T0: Admission (n = 50) T1: T0 + 15 days, or until discharge (n = 17) LOS: median 10 (range: 6–29) days	55 ± 16 years; 48% female	1. Handgrip strength
**Spruit et al. 2003 [51]** **Belgium**	Case control study	Age: NS Condition: COPD Setting: NS	T0: Day 2 (n = 34) T1: Day 7 (n = 25)	69 ± 7 years; % female NS	1. Knee extension strength
**Mets et al. 2004 [41]** **Belgium**	RCT CG: no study medication TG1: NSAID during first 2 weeks of hospitalisation TG2: antipyretic treatment with acetaminophen during first 2 weeks of hospitalisation	Age: ≥ 70 years Condition: Acute infection Setting: Geriatric ward	T0: Day 0–2 (CG: n = 14, TG1: n = 14, TG1: n = 15) T1: T0 + 7 days (CG: n = 14, TG1: n = 14, TG1: n = 15) T2: T0 + 14 days(CG: n = 14, TG1: n = 14, TG1: n = 15)	CG: 84 ± 6 years; 73% female TG1: 85 ± 6 years; 64% female TG2: 85 ± 6 years; 79% female	1. Handgrip strength 2. Handgrip fatigue resistance
**Vermeeren et al. 2004 [42]** **Netherlands**	RCT CG: placebo (flavoured water) TG: nutritional supplements	Age: NS Condition: COPD Setting: NS	T0: Day 1 (CG: n = 24, TG: n = 23) T1: Day 3 (CG: n = 24, TG: n = 23) T2: Day 7 (CG: n = 24, TG: n = 23)	CG: 66 ± 8 years; 25% female TG: 65± 10 years; 64% female	1. Handgrip strength (measured at T0 and T2) 2. Knee extension strength (measured at T1 and T2) 3. Lean body mass (bioelectrical impedance)
**Bautmans et al. 2005 [27]** **Belgium**	Cohort study G1: raised inflammatory markers G2: normal inflammatory markers	Age: NS Condition: NS Setting: Geriatric ward	T0: Day 0 (G1: n = 42, G2: n = 21) T1: T0 + 7 days (G1: n = 39, G2: n = 13) T2: T0 + 14 days (G1: n = 22, G2: n = 5)	G1: 85.8 ± 5.6 years; 67% female G2: 81.0 ± 5.6 years; 67% female	1. Handgrip strength 2. Handgrip fatigue resistance 3. Shoulder extension 4. Hip extension
**Pitta et al. 2006 [28]** **Belgium** **Cohort**	Cohort study	Age: NS Condition: COPD Setting: NS	T0: Day 2 (n = 24) T1: Day 7 (n = 18)	Median 69 (IQR: 60–78) years; 6% female	1. Knee extension strength
**Crul et al. 2007 [52]** **Belgium**	Case control study	Age: NS Condition: COPD Setting: NS	T0: Day 2 (n = 14) T1: Day 7 (n = 14)	68 ± 8 years; 7% female	1. Knee extension strength
**Crul et al. 2010 [53]** **Belgium**	Case control study	Age: NS Condition: COPD Setting: NS	T0: Day 2 (n = 9) T1: Day 7 (n = 9)	67 ± 8 years; 0% female	1. Knee extension strength
**Troosters et al. 2010 [43]** **Brazil**	RCT CG: Usual care TG: Usual care + daily quadriceps resistance training for 7 days	Age: < 85 years Condition: COPD Setting: Respiratory Division	T0: Day 2 (n = 36) T1: Day 7 (n = 36)	CG: 69 ± 7 years; 26.3% female TG: 67 ± 8 years; 23.5% female	1. Knee extension strength
**Beyer et al. 2011 [44]** **Belgium**	RCT CG: placebo TG: NSAID treatment	Age: ≥ 70 years Condition: raised inflammatory marker Setting: Acute Geriatric ward	T0: Day 0–2 (CG: n = 15, TG: n = 15) T1: T0 + 7 days (CG: n = 14, TG: n = 14) T2: T0 + 14 days (CG: n = 13, TG: n = 10) T3: T0 + 21 days (CG: n = 11, TG: n = 8)	CG: median 82.5 (IQR: 79.5–86.5) years; 71.4% female TG: median 85.0 (IQR: 76.8–91.0) years; 64.3% female	1. Lean body mass 2. Handgrip strength 3. Handgrip fatigue resistance 4. Handgrip work
**Wieboldt et al. 2012 [54]** **United Kingdom**	Case control study	Age: adult Condition: Cystic Fibrosis Setting: NS	T0: Day 0–1 (n = 17) T1: Discharge (n = 16) LOS 8.6 ± 3.6 days	29.1 ± 8.6 years; 31% female	1. Handgrip strength 2. Knee extension strength
**Arezzo di Trifiletti et al. 2013 [29]** **Italy**	Cohort study G1: with anorexia G2: without anorexia	Age: NS Condition: NS Setting: Internal Medicine ward	T2: Day 0–1 (G1: n = 11; G2: n = 94) T3: Discharge (G1: n = 11; G2: n = 94) Median LOS G1: 21.8 ± 19.2 days, G2: 13.8± 9.3 days	G1: 72.4 ± 12.9 years; 55% female G2: 65.4 ± 16.6 years; 26.6% female	1. Handgrip strength
**Bodilsen et al. 2013 [30]** **Denmark**	Cohort study	Age: ≥ 65 years Condition: Acute medical illness Setting: NS	T0: Day 0–1 (n = 33) T1: Discharge (n = 23) Median LOS: 7.5 (IQR: 4.25–11.0)	82.7 ± 8.2 years; 46% female	1. Handgrip strength 2. Knee extension strength
**Burtin et al. 2013 [55]** **Belgium**	Case control study	Age: adult Condition: Cystic Fibrosis Setting: NS	T0: Day 0 (n = 19) T1: Day 13 (n = 19)	25 ± 6 years; 32% female	1. Knee extension strength a. Isometric maximum voluntary contraction b. Transcutaneous magnetic twitch stimulation
**Mesquita et al. 2013 [31]** **Brazil**	Cohort study	Age: NS Condition: COPD Setting: NS	T0: Day 0 (n = 19) T1: Discharge (n = 19) Median LOS: 4 (IQR: 3–5)	67 ± 11 years; 36.8% female	1. Knee extension strength
**Borges et al. 2014 [45]** **Brazil**	RCT CG: usual care TG: usual care + resistance training;	Age: NS Condition: COPD Setting: NS	T0: Day 1 (CG: n = 25, TG: n = 21) T1: Discharge (CG: n = 14, TG: n = 15) LOS: CG: 9.6 ±3.2, TG: 8.0 ± 2.2	CG: 67.8 ± 9.0 years; 28.6% female TG: 64.1 ± 12.5 years; 46.7% female	1. Shoulder abduction strength 2. Shoulder flexion strength 3. Elbow flexion strength 4. Knee extension strength 5. Hip flexion strength 6. Knee flexion strength
**Martín-Salvador et al. 2015 [32]** **Spain**	Cohort study G1: <75 years of age G2: ≥ 75 years of age	Age: As grouping categories Condition: Pneumonia Setting: NS	T0: “Admission” (G1: n = 68, G2: n = 48) T1: Discharge LOS: G1: 8.33 ± 3.9 days, G2: 8.05 ± 3.1 days	G1: range 35–72 years; 36.1% female G2: range 75–86 years; 54.2% female	1. Handgrip strength 2. Knee extension strength
**José et al. 2016 [47]** **Brazil**	RCT CG: usual care; TG: usual care + exercise programme	Age: > 18 years Condition: Pneumonia Setting: NS	T0: Day 0–1 (CG: n = 17, TG: n = 32) T1: T0 + 9 days (CG: n = 17, TG: n = 32)	CG: 59 ± 18 years; 41% female TG: 51 ± 21 years; 47% female	1. Elbow flexion strength 2. Shoulder abduction strength 3. Knee extension strength 4. Knee flexion strength
**Martín-Salvador et al. 2016 [48]** **Spain**	RCT CG: usual care TG: usual care + one hour per day of physiotherapy treatment (breathing exercises, electrostimulation and resistance exercises)	Age: 65–90 years Condition: COPD Setting: Respiratory Units	T0: Day 0 (CG: n = 20, TG: n = 24) T1: Discharge (CG: n = 20, TG: n = 24) LOS: CG: 9.49 ± 4.3, TG: 8.39 ± 3.3	CG: 77.4 ± 5.2; 22% female TG: 78.82 (6.3); 16.8% female	1. Knee extension strength
**Rossi et al. 2016 [33]** **Italy**	Cohort study	Age: ≥ 65 years Condition: NS Setting: Geriatrics Division	T0: Day 2 T1: Discharge LOS: 10.54 ± 4.38 days	80.83 ± 7.14 years; 37.1% female	1. Handgrip strength
**Torres-Sánchez et al. 2016 [46]** **Spain**	RCT CG: usual care; TG: usual care + pulmonary rehabilitation (exercise based)	Age: NS Condition: COPD Setting: Respiratory ward	T0: “Admission” (CG: n = 25, TG: n = 24) T1: Discharge (CG: n = 25, TG: n = 24) Minimum LOS 7 days; LOS: CG: 8.8 ± 2 days, TG: 8.7 ± 2 days	CG: 73.70 ± 7.10 years; 9.1% female TG: 72.36 ± 8.91 years; 0% female	1. Handgrip strength 2. Knee extension strength
**Jones et al. 2017 [34]** **United Kingdom**	Cohort study	Age: ≥ 65 Condition: NS Setting: Older Persons’ Unit	T0: Day 0–1 (n = 75) T1: Discharge (n = 75) Median LOS: 14 (IQR: 9–26) days	84.77 ± 7.06 years; 64% female	1. Handgrip strength
**Karlsen et al. 2017 [35]** **Denmark**	Cohort study	Age: ≥ 65 years Condition: NS Setting: Acute Geriatric Department	T0: Day 1–3 (n = 151) T1: Day 4–7 (n = 122) T2: Day 8–12 (n = 104)	85.2 ± 7.2 years; 74% female	1. Handgrip strength
**Matsuo et al. 2017 [36]** **Japan**	Cohort study	Age: ≥ 65 years Condition: NS Setting: Acute Care wards	T0: Day 0–1 T1: Discharge Median LOS 26 (IQR:16–40) days	80.5 ± 7.9 years; 56.3% female	1. Handgrip strength
**Norheim et al. 2017 [37]** **Denmark**	Cohort study G1: Persistent inflammation during admission G2: Inflammation at admission resolved	Age: NS Condition: Acute illness with raised inflammatory markers Setting: Geriatric ward	T0: “Admission” (G1: n = 138; G2: n = 76) T1: Discharge (G1: n = 138; G2: n = 76) LOS G1: median 11.0 (IQR: 8.0–15.0) days, G2: 8.0 (6.0–11.0) days	G1: median 87 (IQR: 81–91) years; 67% female G2: 85 (79–91) years; 68% female	1. Handgrip strength
**Torres-Sánchez, Valenza et al. 2017 [49]** **Spain**	RCT CG: usual care; TG: usual care + seated pedalling programme	Age: ≥ 65 years Condition: COPD Setting: Respiratory ward	T0: “Admission” (CG: 29; TG: 29) T1: Discharge (CG: 29; TG: 29) LOS: CG: 10.38 ± 2.47, TG: 12.47 ± 1.9	CG: 72.12 ± 8.19 years; 31% female TG: 75.65± 6.25 years; 24% female	1. Knee extension strength
**Torres-Sánchez, Cabera-Martos et al. 2017 [38]** **Spain**	Cohort study	Age: > 40 years Condition: COPD Setting: Respiratory Care Unit	T0: Day 1 (n = 52) T1: Discharge (n = 52) LOS: 12.17 ± 5.17 days	75.87 ± 6.14 years; 4.5% female	1. Handgrip strength 2. Knee extension strength

Abbreviations: SD: standard deviation; NS: not specified; LOS: length of hospital stay; RCT: Randomised Control Trial; CG: Control Group; TG: Treatment Group; COPD: Chronic Obstructive Pulmonary Disease; NSAID: non-steroidal anti-inflammatory drug.

### Change in upper limb strength in those receiving ‘usual care’

**Fig 2:**
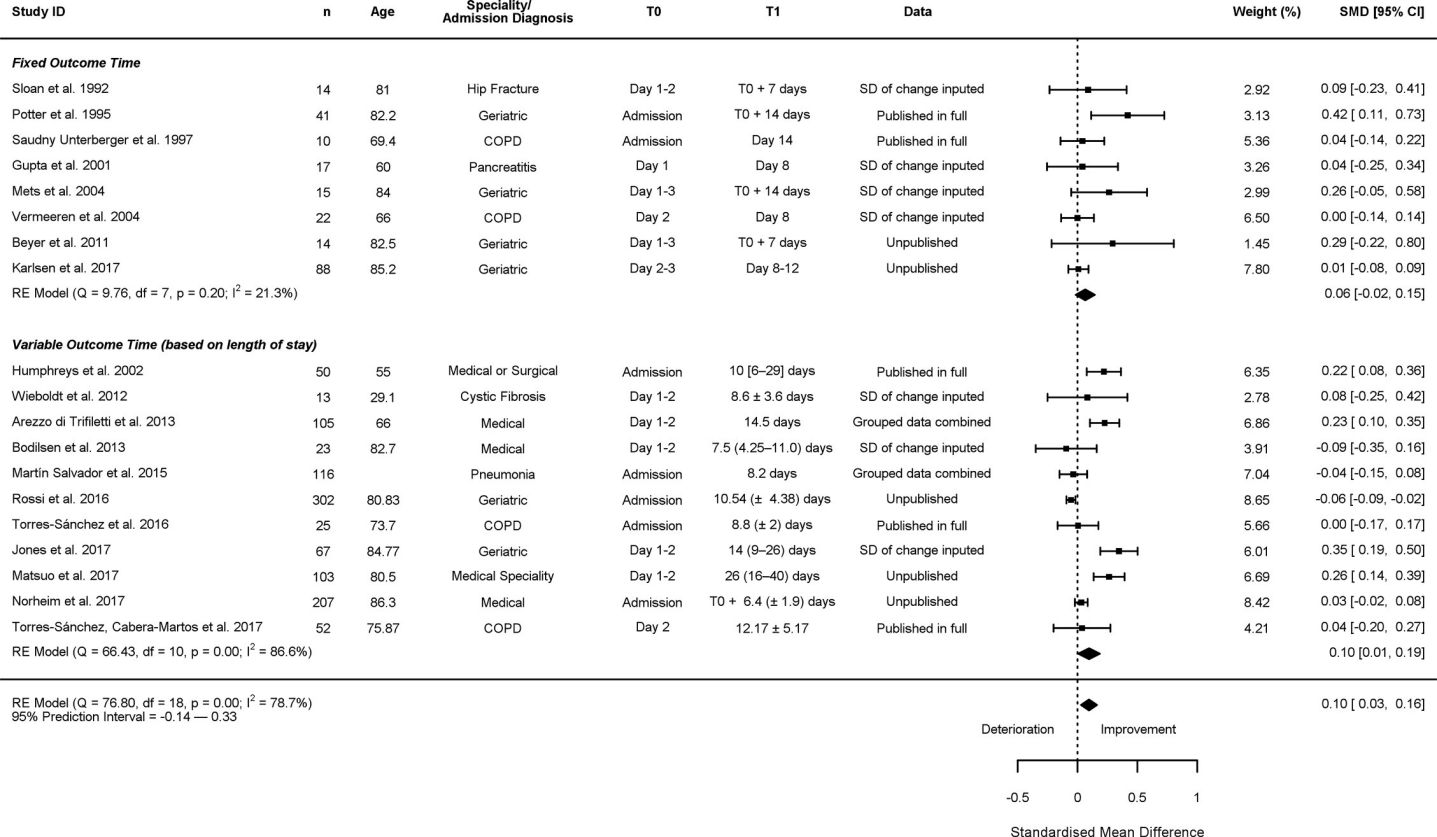
Change in grip strength. In column ‘T1’ presented as mean (± SD) or median (IQR) or median [range]. NR: Not reported; COPD = Chronic obstructive pulmonary disease.

Twenty studies reported change in grip strength [23, 26, 27, 29, 30, 32–42, 44, 46, 50, 54]; 10 of these studies reported change based on fixed time points, and 10 reported change over the patients’ length of stay in hospital. One study was excluded from the meta-analysis as they presented insufficient data, but reported non-significant changes in grip strength over the first 2 weeks of hospitalisation [27]. Meta-analysis of change between baseline and follow-up in a random effects model calculated an average increase of grip strength, SMD = 0.10 (95% CI: 0.03; 0.16) (Fig 2). There was large between-study heterogeneity in changes in grip strength (I^2^ = 78.66%), with 95% prediction intervals (95% PI): -0.14; 0.33, suggesting that although on average grip strength improved, in some settings or populations it may deteriorate or remain unchanged. Sub-group analysis of the studies that used a fixed time also calculated an average increase in grip strength; there was less heterogeneity, and results were non-significant: SMD = 0.06 (95% CI: -0.02; 0.15), I^2^ = 21.30%, 95% PI: -0.06; 0.19. Finally, meta-regression showed that age did not account for any of the heterogeneity seen in the primary analysis of grip strength (k = 18, R^2^ = 0.00%, ß = 0.00 (95% CI -0.01; 0.01), p = 0.956).

Three studies measured grip fatigue resistance (i.e. time that the patient is able to maintain a maximum voluntary contraction before the force produced diminishes by more than 50%) [27, 41, 44]. Insufficient data was reported for meta-analysis. Mets et al. [41] and Beyer et al. [44] reported no significant change in their control/placebo groups. Bautmans et al. [27] reported a significant improvement in grip fatigue resistance only in the patients with non-elevated levels of CRP or fibrinogen on admission to hospital. Beyer et al. [44] also measured the variable ‘grip work’ calculated as: (grip strength*0.75)*fatigue resistance, and there was no significant change in their placebo group.

Two studies measured change in shoulder abductor strength and elbow flexor strength [45, 47], one study measured change in shoulder flexion [45], and one study change in shoulder extension strength [27]. None found significant difference in participants receiving usual care (this was confirmed with the José et al. [47] as within subject change was not reported in the original manuscript).

### Change in lower limb strength in those receiving ‘usual care’

**Fig 3:**
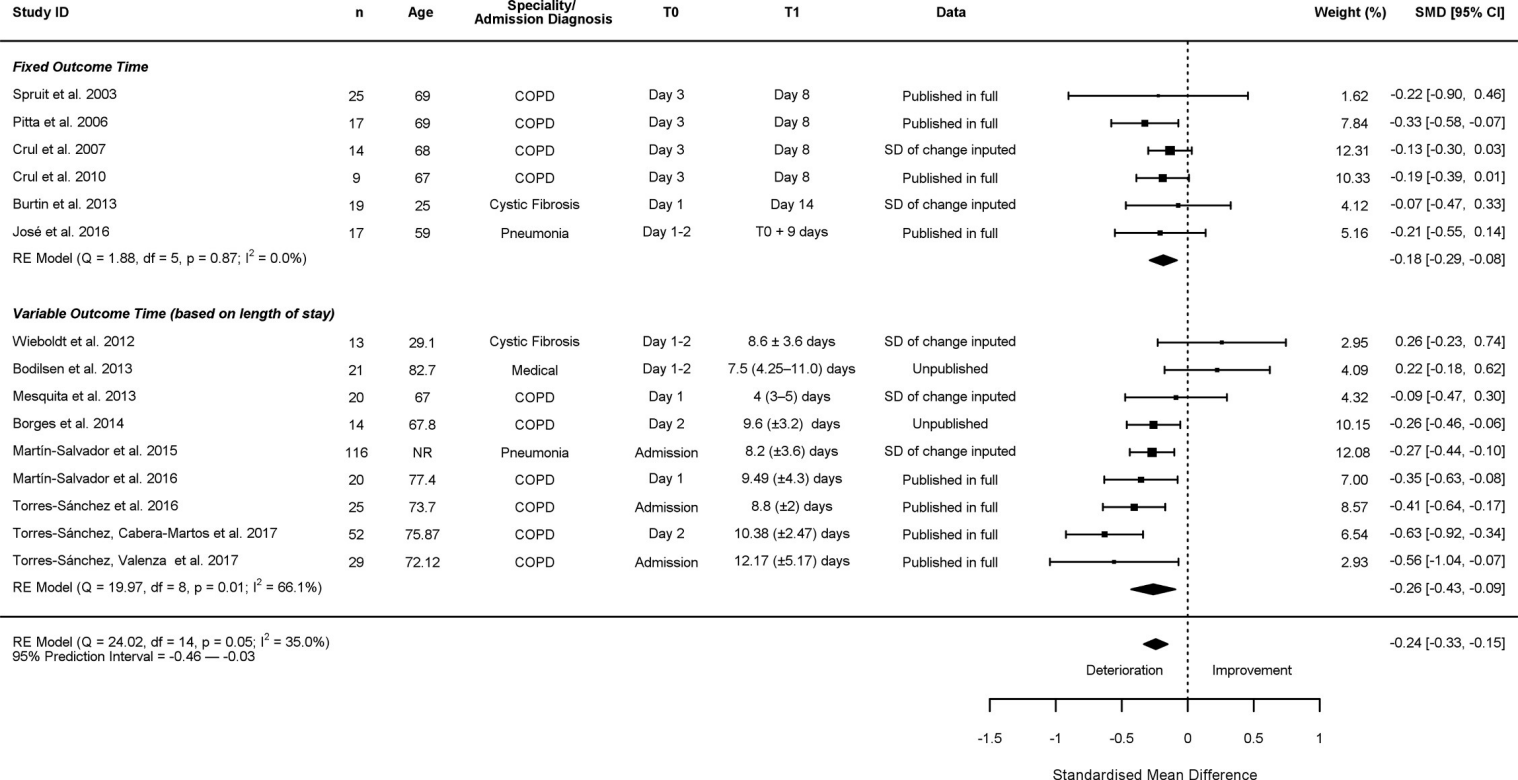
Change in knee extension strength. In column ‘T1’ presented as mean (± SD) or median (lower quartile–upper quartile). COPD = chronic obstructive pulmonary disease, NR = not reported.

Of 17 studies measuring change in knee extension strength whilst in hospital [28, 30–32, 38, 42, 43, 45–49, 51–55], 15 were included in the meta-analysis. Two studies were excluded from meta-analysis, one due to insufficient data reporting [43], and the other due to the baseline measurement being outside the 72 hours cut-off point [42], and neither found significant changes. Of the studies included in the meta-analysis, 6 used fixed time points for T1, 9 used variable time points. There was a reduction in knee extension strength from T0 to T1, SMD = -0.24 (95% CI: -0.33; -0.15) (Fig 3). There was significant between study heterogeneity in changes in knee extension strength (I^2^ = 34.97%, 95% PI -0.46; -0.03). In the sub-analysis of the studies using fixed outcome points the overall SMD was calculated as -0.18 (95% CI: -0.29; -0.08, I^2^ = 0.00%, 95% PI: -0.29; -0.08). The intervals between T0 and T1 in the subgroup ranged from 5 to 13 days. Finally, meta-regression showed that age did not significantly account for heterogeneity seen in the primary model of change in knee strength (k = 15, R^2^ = 27.67%, ß = -0.01 (95% CI -0.01; 0.00), p = 0.099).

Two studies measured knee flexor strength, and neither found significant changes in participants receiving usual care [45, 47]. Finally, Borges et al. [45] reported a significant loss in hip flexor strength of approximately 10% between admission and discharge.

### Change in muscle mass

**Fig 4:**
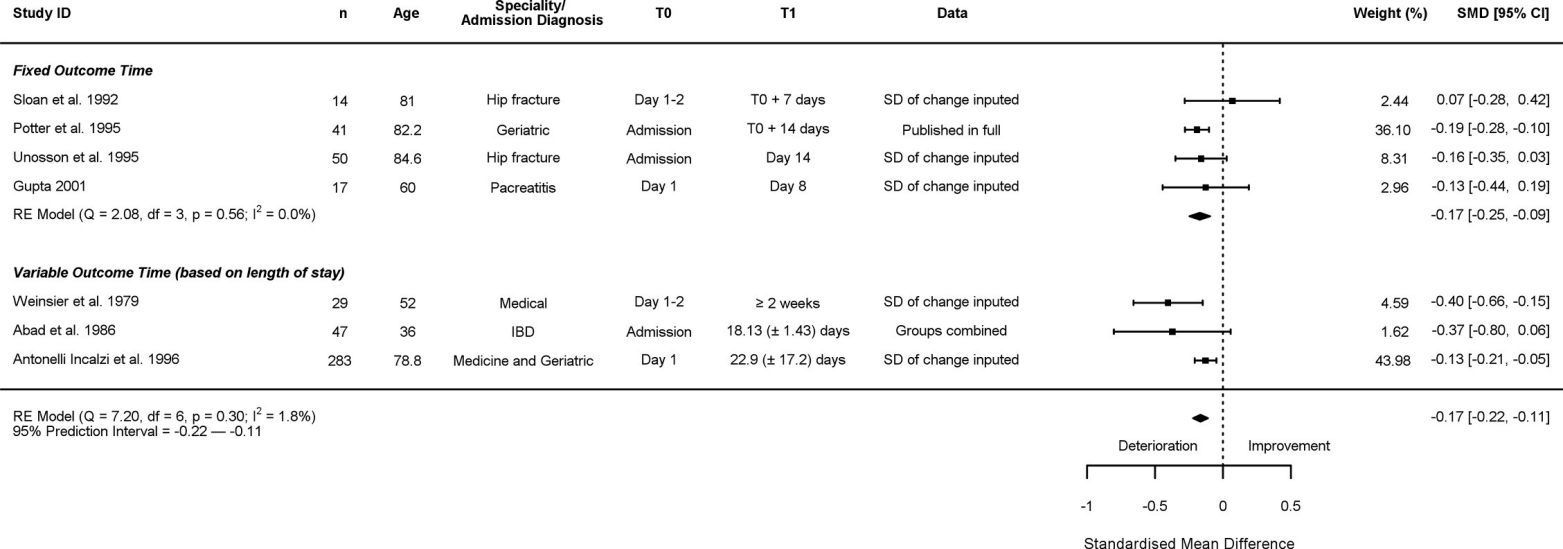
Change in mid-arm muscle mass. In column ‘T1’ presented as mean (± SD). IBD = Inflammatory Bowel Disease.

Nine studies measured muscle mass [21–26, 39, 42, 44]. Seven measured mid arm muscle circumference [21–26, 39], two studies used bio-impedance [39, 42], and one used naturally occurring isotopic K in a whole body counter to measure lean body mass [44]. Of the studies measuring mid-arm muscle circumference, the estimated SMD of change was -0.17 (95% CI: -0.22; -0.11) (Fig 4). There was minimal between study heterogeneity in changes in mid-arm muscle mass: I^2^ = 1.81%, 95% PI: -0.22; -0.11. The sub-analysis of 4 studies using fixed outcome points had a SMD of change of -0.17 (95% CI: -0.25; -0.09, I^2^ = 0.01%, 95% PI: -0.25; -0.09). The intervals between T0 and T1 in the subgroup ranged from 7 to 14 days. The studies using bio-impedance and a whole body counter all reported no significant change during hospitalisation [39, 42, 44].

### Association with change in muscle and markers of inflammation

Four studies measured the association between change in muscle strength and inflammation [33, 37, 47, 51]. Three studies measured the association between C-reactive protein (CRP) and muscle strength. José et al. [47] reported no association between CRP and any other variables. Bautmans et al. [27] reported no association between grip strength or grip fatigue resistance and CRP, but significant negative correlations between CRP and shoulder extension strength and hip extension strength. Rossi et al. [33] reported a significant association between change in handgrip strength and CRP. Norheim et al. [37] recruited patients with raised CRP, and reported that handgrip strength only improved in patients whose CRP reduced to below 10mg·L^-1^ after 1 week of hospitalisation; they did not however find a significant correlation between change in CRP and change in handgrip strength.

Three studies reported significant negative correlations between Interleukin 6 (IL-6) and muscle strength, specifically, grip strength [27], grip fatigue resistance [27, 41], shoulder extension strength [27], hip extension [27], and knee extension strength [51]. Spruit et al. [51] also reported a negative correlation with Interleukin 8 (IL-8) and knee extension strength. Crul et al. [52] reported no correlation between IL-6 and IL-8 with knee extension strength.

Beyer et al. [44] measured 25 different cytokines/chemokines, and reported multiple significant negative correlations with baseline cytokines/chemokines and grip strength and grip fatigue resistance (though not with IL-6 or IL-8) in the placebo group.

### Association with change in muscle strength and physical activity in hospital

Three studies reported on the relationship between level of activity during hospitalisation and muscle strength [28, 30, 55]. Pitta et al. [28] reported that the time spent in weight-bearing activities was positively correlated to quadriceps force at day 8 of the hospitalisation period (*r* 0.47; p 0.048). Bodilsen et al. [30] reported no significant associations between time spent standing or walking during hospitalisation and changes in knee-extension strength or functional performance during hospitalisation (P = 0.849). Burtin et al. [55] reported that individual changes in twitch force during antibiotic therapy were strongly correlated with daily time spent in activities of at least moderate intensity; no relationship was found with the twitch force and daily number of steps, or knee extension strength and moderate intensity activity or daily number of steps [55].

### Effect of interventions

Eleven RCTs were included [39–49]; 5 examined the effect of an exercise based intervention [43, 45–47, 49]; two the effect of a nutritional intervention [40, 42], and Martín-Salvador et al. [48] a combination of physiotherapy led breathing exercises, electrical stimulation and resistance exercises. Sloan et al. [39] investigated the safety of giving anabolic steroids to older patients with hip fractures. Mets et al. [41] and Beyer et al. [44] investigated the effect of giving non-steroidal anti-inflammatories on patients admitted to a geriatric ward with inflammation of acute infectious origin.

**Fig 5:**
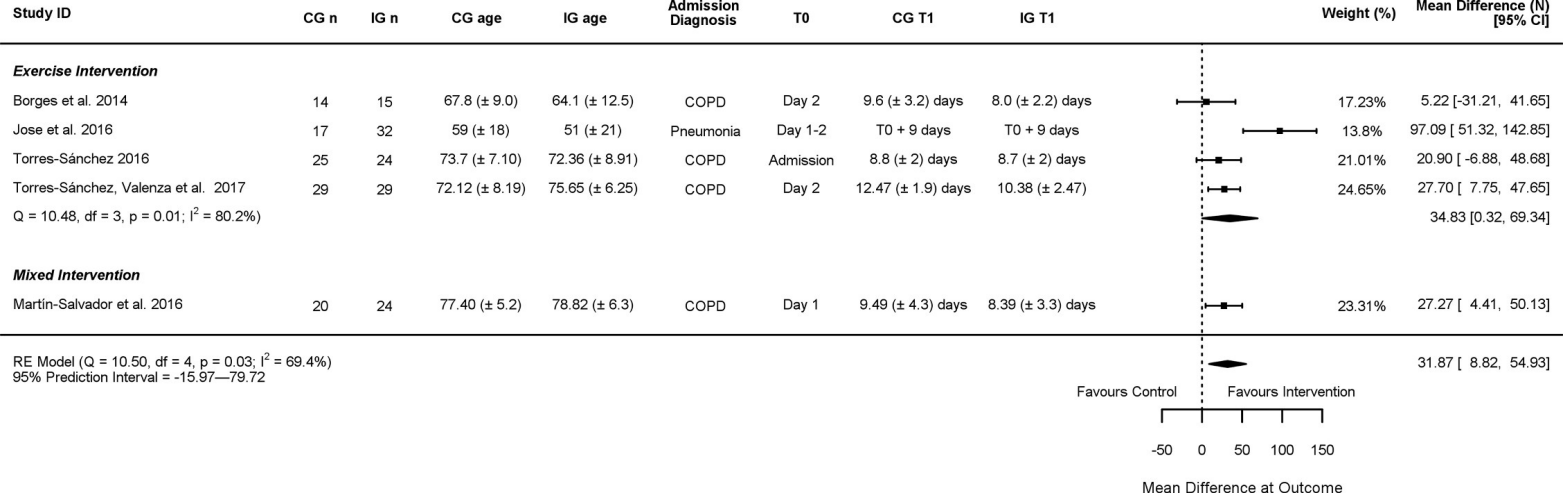
Effectiveness of exercise and physiotherapy based interventions. CG = control group, IG = intervention group, N = newtons.

The studies investigating the effect of exercise and physiotherapy based interventions all found significant between group differences in the muscle strength outcomes in favour of the treatment group [43, 45–49]. The meta-analysis of the exercise and physiotherapy based interventions demonstrated a favourable mean difference (MD) however high heterogeneity, MD = 31.87 (95% CI: 8.82; 54.93) I^2^ = 69.4 (95% PI: -15.97; 79.72) (Fig 5). The study by Troosters et al. [43] could not be included in the meta-analysis due to insufficient data, but also reported a trend towards significant improvement in the exercise group knee extension force: 9.7 ± 16% compared to -1 ± 13% p = 0.05.

The two studies investigating nutritional interventions found no effect in terms of or grip strength [40, 42], or quadriceps strength (day 4 to day 8) [42].

Sloan et al. [39] found no effect of giving anabolic steroids to older patients with hip fractures; however, the primary aim of their pilot study was to assess the safety, and investigating effect was a secondary aim.

Mets et al. [41] found significant improvement in patients receiving NSAID compared to the group receiving acetaminophen and the control group in grip fatigue resistance but not in grip strength or mobility. Beyer et al. [44] found no difference between the groups receiving NSAID treatment and a placebo treatment in terms of grip strength or grip fatigue resistance.

### Publication bias

**Fig 6:**
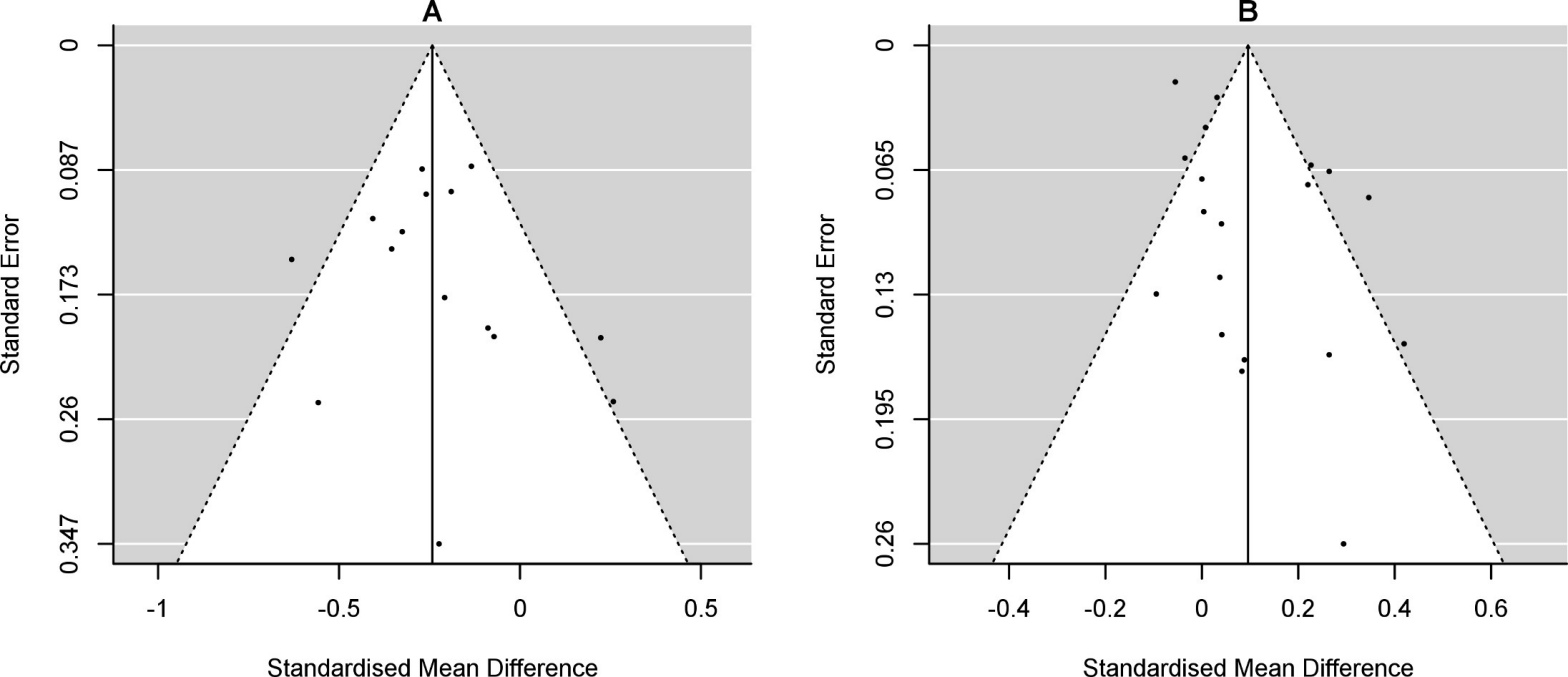
**A: Funnel plots.** A: Change in grip strength; B: Change in knee extension strength.

Funnel plots for all meta-analyses are presented in Fig 6. There was no evidence of publication bias in meta-analysis of change in grip strength: z = 1.373, p = 0.170, or change in knee extension strength: z = 0.824, p = 0.410.

## Discussion

### Summary of main findings

Our results suggest that there is significant heterogeneity in changes in skeletal muscle during unplanned hospital admissions in adults. On average, the studies included in this review indicated that although on average grip strength seemed to have a modest improvement, there was large heterogeneity, and the observed change when using a fixed time point showed that this was a non-significant finding. The included studies did however show small reductions in knee extension strength and muscle mass, which were consistent across studies when a fixed time point (between 5 and 14 days) for outcome was used. The clinical importance of these changes are likely to vary between patients with regards to their pre-morbid physiological and functional reserves.

Fixed time points ensure that all participants have experienced the same level of exposure to hospitalisation. They are also likely to reduce some of the heterogeneity in the study populations, for example, they are likely to include fewer ‘fitter’ patients or those with low illness severity who are discharged prior to T1. It is also possible that the studies using variable time points may be effected by a regression towards the mean effect in those participants who experience a very long hospital stay, that is those with high initial strength may be more likely to deteriorate, and those with low initial strength more likely to improve.

Age did not significantly account for the heterogeneity seen in change in grip strength or knee extension strength. This may be surprising given the supposed physical vulnerability and frailty of many older patients, and it may be expected that they would have greater muscle loss whilst in hospital. However, this may be explained by younger patients on average having greater muscle strength and mass at baseline, and therefore having the most to lose. It may also be clinically relevant to consider threshold levels of muscle strength and mass, that is, even a relatively large loss of strength or mass in a previously fit and strong individual may not impact on their ability to perform basic activities of daily living independently.

The degree and duration of inflammation appeared to correlate with muscle strength and change in muscle strength, although this was not a consistent finding across studies or between different inflammatory markers. Some of the discrepancy may be explained by low sample sizes in some of the studies. With regards to IL-6, Crul et al. [52] and Beyer et al. [44] were the 2 studies to find no correlation between strength and IL-6, but both had relatively low numbers of participants. Crul et al. [52] included 14 hospitalised patients, and Beyer et al. [44] had 14 participants in their control group. The findings of a correlation between inflammation (particularly elevated cytokines) and muscle weakness are consistent with the understanding of critical care or sepsis acquired muscle weakness [56].

A correlation between the level or amount of activity and knee extension strength at T1 was found, but not with the level of activity and change in knee-extension strength between T0 and T1. This may indicate that other factors such as inflammation are more responsible for changes in muscle strength, but due to the low sample sizes and number of studies examining this relationship it is not thought possible to draw any firm conclusions.

Exercise and physiotherapy interventions appeared to have a beneficial effect on knee extension strength, but there was large heterogeneity in the meta-analysis despite all studies focusing on respiratory conditions. Much of the heterogeneity is likely due to differences in the interventions. For instance, the frequency of exercise varied from twice daily [46] to a minimum of 3 sessions during the hospital admission [45], and the type of exercise also varied; 2 studies focused on resistance training [45, 47], and one on multi-modal pulmonary rehabilitation [49]. The biggest benefit was seen in José et al. [47] who included a younger population, and provided daily resistance training at 70% of a 1 repetition maximum effort contraction, and it was also the only study to use fixed outcome time points.

### Comparison with other reviews

The findings of the grip-strength and mid-arm muscle circumference meta-analysis differ slightly with those of Van Ancum et al. [57]; this may be due to a larger number of studies included in our review, although we had different inclusion criteria and used different meta-analysis methods, including for calculating SMD. The findings of loss of muscle mass and knee extension strength are consistent with the findings of Alley et al. [58]. The reason why grip strength showed a trend towards improvement whereas knee extension strength declined is unclear, but may be due to the effect of bed rest that is known to have a greater effect on lower limb muscle groups than upper limbs [59]. It is likely that both grip strength and knee extension strength are initially negatively affected by inflammation or the acute stressor causing hospital admission, and whereas grip strength returns towards a pre-illness level (and thereby demonstrates improvement), knee extension strength is further affected by immobility and reduced level of activity as seen in studies of the effect of bed rest in healthy volunteers [5]. It is also worth noting that the pattern of greater proximal weakness (knee extension) than distal weakness (grip strength) is seen in critical care myopathy [60]. It may be that the results observed within the review represent a milder form of critical care myopathy. It is also possible that the mechanisms responsible for critical care myopathy explain why the proximal measure of mid-arm muscle mass reduced but the distal measure of grip strength did not. Indeed, research on healthy individuals during periods of immobilisation usually shows a relatively larger reduction in muscle strength compared to mass (of the same muscle group) [61, 62]. It seems appropriate therefore to assume that there are greater losses to proximal muscle than distal in most hospitalised adults. Some caution must be shown to interpreting change in mid-arm muscle circumference during hospitalisation though as it may be influenced by factors such as oedema.

### Limitations of included studies

The main limitation observed when assessing risk of bias was that studies assessing outcomes at admission and discharge did not adjust for difference in length of stay. Few studies reporting muscle strength as a primary outcome adjusted for possible confounding variables, such as illness severity, age, premorbid level of function, and none of the RCTs reported on compliance with the intervention. Many of the studies that investigated relationships between change in muscle strength and participant characteristics had small sample sizes, limiting the validity of the findings. There was a lot of inconsistency with descriptions of usual care and interventions (S3 Table), such as access to and content of physiotherapy and occupational therapy interventions, or nutritional intake.

### Limitations of this review

A major limitation of the meta-analyses is the potential confounding introduced through low quality observational studies. As such we have avoided suggesting that degree of muscle change during a period of hospitalisation can be predicted, or the clinical implications the observed changes may have, other than to suggest the expected direction of change, and that the clinical significance is dependent on pre-morbid physiological reserves. Further, causality cannot be inferred. In addition, the review is limited by the fact that we only included studies published in English.

The review included a relatively heterogeneous sample of studies in terms of reason for admission, age, intervals between baseline measurements and outcome as well as the likely differences in hospital care over time and between countries. Importantly however, the measures of heterogeneity in the meta-analysis showed minimal differences between changes when the T1 time point was fixed. This suggests that length in hospital (degree of exposure) may be the most important confounding factor in explaining change in skeletal muscle.

The review did not put the change in muscle strength or mass within the context of pre-illness levels. It is likely that the T0 muscle strength measurement represented an already reduced level of strength compared to pre-admission levels. This is supported by the negative correlation observed between admission inflammation levels and muscle strength [63]. As well as pre-hospital inflammation affecting muscle, there may also be confounding from pre-admission immobility. Reduction in knee extension strength during hospitalisation may therefore underestimate total loss of strength associated with a period of unplanned hospitalisation.

Finally, using methods to impute some data rather than having access to the original data may have been another possible source of bias within the meta-analysis. We conducted a sensitivity analysis to examine whether using medians and interquartile ranges to estimate the mean and standard deviations introduced bias (see S4 Table), and found minimal differences to the estimated SMDs and 95% CIs.

## Conclusion

The review suggests that adults who undergo an unplanned hospital admission may experience a small reduction in knee extension strength and mid-arm muscle mass. There was weak evidence that grip strength may improve marginally in certain populations; however, when measured over a fixed time point, this was not a significant increase. Inflammation appeared to be associated with higher loss of muscle strength during hospitalisation. There was inconclusive evidence that the level of physical activity during hospitalisation affects change in skeletal muscle. Exercise-based interventions appeared beneficial in improving/maintaining knee extension strength.

Prospective research is needed to clarify the contribution of confounding factors underlying the observations reported in this review. Of particular interest would be the contributions of potentially modifiable factors such as levels of physical activity during hospitalisation, and the influence of potentially modifiable patient-related factors as well as factors related to the environment and processes of hospital care.

## Supporting information

S1 Text MEDLINE via OVID search strategy. (DOCX)

S1 Table Risk of bias with Downs and Black tool. Abbreviations: Y = Yes, N = No, U = Unable to determine *Modified item, reviewers asked the following question: Did the study have sufficient power to detect a clinically important effect? (Yes/No/Unable to determine).
(DOCX)

S2 Table Risk of bias with NIH checklist for observational cohort and cross-sectional studies. Abbreviations: Y = Yes, N = No, NA = Not applicable, NR = Not reported, CD = Cannot determine.
(DOCX)

S3 Table Description of usual care and interventions of included randomised control trials. (DOCX)

S4 Table Sensitivity analysis examining effect of excluding studies that reported results as median and inter-quartile range. Abbreviations: MAMC = Mid arm muscle circumference.
(DOCX)

S5 Table Data used in meta-analysis. (DOCX)

S6 Table PRISMA checklist. (DOC)
