# Supplementary material for: Change in skeletal muscle associated with unplanned hospital admissions in adult patients: A systematic review and meta-analysis
Source: PLoS One. 2019 Jan 4;14(1):e0210186. doi: 10.1371/journal.pone.0210186 (PMC6319740; doi:10.1371/journal.pone.0210186)
Supplement: S1 Text — (DOCX) [file pone.0210186.s001.docx]

**S1 Text: MEDLINE via OVID search strategy**

(((muscle* or quadricep* or (vastus adj lateralis) or (vastus adj medialis) or gluteal or buttock* or soleus or hamstring* or gastrocnemius or (rectus adj femoris)) adj3 ((strength* or power* or force* or (cross adj section*) or CSA or or depth or change* or diameter* or circumference or dynamomet* or dynapeni* or sarcopeni* or atroph* or or hypertroph* or hypotroph* or decondition* or waste* or wastage* or loss* or lost*)ti,ab. **or** **(**(handgrip strength) or (handgrip strength) or (muscle strength)**)** or exp muscle atrophy/ or exp sarcopenia/)) **and** ((hospitalis* or hospitaliz* or inpatient* or in-patient* or (length* adj3 stay*) or inpatient*).ti,ab. Or exp hospitalization/ or exp inpatients/ or exp "Length of Stay"/)
