# Supplementary material for: Change in skeletal muscle associated with unplanned hospital admissions in adult patients: A systematic review and meta-analysis
Source: PLoS One. 2019 Jan 4;14(1):e0210186. doi: 10.1371/journal.pone.0210186 (PMC6319740; doi:10.1371/journal.pone.0210186)
Supplement: S1 Table — Abbreviations: Y = Yes, N = No, U = Unable to determine *Modified item, reviewers asked the following question: Did the study have sufficient power to detect a clinically important effect? (Yes/No/Unable to determine). (DOCX) [file pone.0210186.s002.docx]

**S1 Table: Risk of bias with Downs and Black tool**

|  | **Checklist Item** | | | | | | | | | | | | | | | | | | | | | | | | | | |
| --- | --- | --- | --- | --- | --- | --- | --- | --- | --- | --- | --- | --- | --- | --- | --- | --- | --- | --- | --- | --- | --- | --- | --- | --- | --- | --- | --- |
| **Study ID** | **1** | **2** | **3** | **4** | **5** | **6** | **7** | **8** | **9** | **10** | **11** | **12** | **13** | **14** | **15** | **16** | **17** | **18** | **19** | **20** | **21** | **22** | **23** | **24** | **25** | **26** | **27*** |
| **Sloan et al. 1992[39]** | Y  1 | Y  1 | Y  1 | Y  1 | P  1 | Y  1 | Y  1 | Y  1 | Y  1 | N 0 | N 0 | U 0 | Y1 | Y 1 | U 0 | Y 1 | Y 1 | Y 1 | U 0 | Y 1 | Y 1 | Y 1 | Y 1 | U 0 | U 0 | Y 1 | U 0 |
| **Saudny-Unterberger 1997[40]** | Y  1 | Y  1 | Y  1 | Y  1 | P  1 | Y  1 | Y  1 | N  0 | N  0 | Y  1 | Y  1 | U  0 | Y  1 | N  0 | Y  1 | Y  1 | Y  1 | Y  1 | U  0 | Y  1 | Y  1 | Y  1 | Y  1 | N  0 | N  0 | N  0 | U  0 |
| **Mets et al. 2004[41]** | Y 1 | Y 1 | Y 1 | Y 1 | P 1 | N  0 | N  0 | Y 1 | Y 1 | N 0 | U 0 | U 0 | Y 1 | N 0 | Y 1 | Y 1 | Y 1 | Y 1 | U 0 | Y 1 | Y 1 | Y 1 | Y 1 | U 0 | Y 1 | Y 1 | Y 1 |
| **Vermeeren et al. 2004[42]** | Y 1 | Y 1 | Y 1 | Y 1 | P 1 | Y 1 | Y 1 | Y 1 | Y 1 | Y 1 | Y 1 | U 0 | Y 1 | Y 1 | Y 1 | Y 1 | Y 1 | Y 1 | Y 1 | Y 1 | Y 1 | Y 1 | Y 1 | U 0 | Y 1 | Y 1 | N  0 |
| **Troosters et al. 2010[43]** | Y 1 | Y 1 | Y 1 | Y 1 | P 1 | Y 1 | Y 1 | Y 1 | Y 1 | Y 1 | U 0 | U 0 | Y  1 | N 0 | N 0 | Y 1 | Y 1 | Y 1 | Y 1 | Y 1 | Y 1 | Y 1 | Y 1 | N 0 | Y 1 | Y 1 | Y 1 |
| **Beyer et al. 2011[44]** | Y 1 | Y 1 | Y 1 | Y 1 | P 1 | Y 1 | Y 1 | Y 1 | Y 1 | Y 1 | Y 1 | U 0 | Y 1 | Y 1 | Y 1 | Y 1 | Y 1 | Y 1 | Y 1 | Y 1 | Y 1 | Y 1 | Y 1 | U 0 | Y 1 | Y 1 | Y 1 |
| **Borges et al. 2014[45]** | Y  1 | Y  1 | Y  1 | Y  1 | P  1 | Y  1 | Y  1 | Y  1 | Y  1 | Y  1 | Y  1 | Y  1 | Y  1 | N  0 | Y  1 | Y  1 | N  0 | Y  1 | U 0 | Y  1 | Y  1 | Y  1 | Y  1 | N  0 | N  0 | Y  1 | Y  1 |
| **José et al. 2016[47]** | Y  1 | Y  1 | Y  1 | Y  1 | Y  1 | Y  1 | Y  1 | Y 1 | Y 1 | Y 1 | U  0 | U  0 | Y  1 | U  0 | Y  1 | Y  1 | Y  1 | Y  1 | U 0 | Y  1 | Y  1 | Y  1 | Y  1 | U  0 | N  0 | Y  1 | Y  1 |
| **Martín Salvador et al. 2016[48]** | Y 1 | Y 1 | Y 1 | Y 1 | P 1 | Y 1 | Y 1 | Y 1 | Y 1 | Y 1 | U 0 | U 0 | Y  1 | N 0 | Y 1 | Y 1 | Y 1 | Y 1 | U 0 | Y 1 | Y 1 | Y 1 | Y 1 | Y 1 | Y 1 | Y 1 | Y 1 |
| **Torres-Sánchez**  **2016[46]** | Y  1 | Y  1 | Y  1 | Y  1 | P  1 | Y  1 | Y  1 | Y  1 | Y  1 | Y  1 | U  0 | U  0 | Y  1 | N  0 | U  0 | Y1 | N  0 | Y  1 | U 0 | Y  1 | Y  1 | Y  1 | Y  1 | N  0 | N  0 | Y  1 | Y  1 |
| **Torres-Sánchez, Valenza et al. 2017[49]** | Y  1 | Y  1 | Y  1 | Y  1 | P  1 | Y  1 | Y  1 | Y  1 | Y  1 | Y  1 | U  0 | U  0 | Y  1 | N  0 | Y 1 | Y  1 | N  0 | Y  1 | U 0 | Y  1 | Y  1 | Y  1 | Y  1 | N  0 | N  0 | Y  1 | Y  1 |
