# Supplementary material for: Change in skeletal muscle associated with unplanned hospital admissions in adult patients: A systematic review and meta-analysis
Source: PLoS One. 2019 Jan 4;14(1):e0210186. doi: 10.1371/journal.pone.0210186 (PMC6319740; doi:10.1371/journal.pone.0210186)
Supplement: S2 Table — Abbreviations: Y = Yes, N = No, NA = Not applicable, NR = Not reported, CD = Cannot determine. (DOCX) [file pone.0210186.s003.docx]

**S2 Table: Risk of bias with NIH checklist for observational cohort and cross-sectional studies.**

|  | **Checklist Items** | | | | | | | | | | | | | |
| --- | --- | --- | --- | --- | --- | --- | --- | --- | --- | --- | --- | --- | --- | --- |
| **Study ID** | **1** | **2** | **3** | **4** | **5** | **6** | **7** | **8** | **9** | **10** | **11** | **12** | **13** | **14** |
| **Weinsier et al. 1979[21]** | Y | Y | CD | Y | N | NA | Y | Y | Y | NA | Y | NA | N | N |
| **Abad et al. 1986[22]** | Y | Y | CD | Y | N | NA | Y | N | Y | NA | Y | NA | Y | N |
| **Potter et al. 1995[23]** | Y | Y | CD | Y | N | NA | Y | Y | Y | NA | Y | NA | Y | N |
| **Unosson et al. 1995[24]** | Y | Y | Y | Y | N | NA | Y | N | Y | NA | Y | NA | Y | N |
| **Antonelli**  **Incalzi et al. 1996[25]** | Y | Y | CD | Y | NR | NA | Y | N | Y | NA | Y | NA | Y | Y |
| **Gupta 2001** | Y | Y | Y | Y | N | NA | Y | N | Y | NA | Y | NA | CD | N |
| **Humphreys 2002[50]** | Y | Y | CD | Y | N | NA | Y | N | Y | NA | Y | NA | N | N |
| **Spruit et al. 2003[51]** | Y | Y | CD | CD | N | NA | Y | Y | Y | NA | Y | NA | N | N |
| **Bautmans et al. 2005[27]** | Y | Y | CD | CD | NR | NA | Y | Y | Y | NA | Y | NA | Y | Y |
| **Pitta et al 2006[28]** | Y | Y | Y | Y | N | NA | Y | N | Y | NA | Y | NA | N | N |
| **Crul et al. 2007[52]** | Y | Y | CD | CD | NR | NA | Y | Y | Y | NA | Y | NA | Y | Y |
| **Crul et al. 2010[53]** | Y | Y | CD | CD | NR | NA | Y | Y | Y | NA | Y | NA | Y | Y |
| **Wieboldt et al. 2012** | Y | Y | CD | CD | NR | NA | Y | N | Y | NA | Y | NA | Y | Y |
| **Arezzo di Trifiletti et al. 2013[29]** | Y | Y | Y | Y | NR | NA | Y | N | Y | NA | Y | NA | Y | Y |
| **Burtin 2013[55]** | Y | Y | CD | CD | N | NA | Y | N | Y | NA | Y | NA | Y | Y |
| **Bodilsen et al. 2013[30]** | Y | Y | Y | Y | Y | NA | Y | N | Y | NA | Y | NA | N | N |
| **Mesquita et al. 2013[31]** | Y | Y | CD | CD | Y | NA | Y | N | Y | NA | Y | NA | Y | N |
| **Martín-Salvador et al. 2015[32]** | Y | Y | CD | Y | N | NA | Y | N | Y | NA | Y | NA | N | N |
| **Rossi 2016** | Y | Y | Y | Y | N | NA | Y | Y | Y | NA | Y | NA | N | Y |
| **Jones et al. 2017[34]** | Y | Y | Y | Y | NR | NA | Y | N | Y | NA | Y | NA | Y | Y |
| Karlsen et al. 2017[35] | Y | Y | CD | Y | Y | NA | Y | Y | Y | NA | Y | NA | Y | Y |
| **Matuso et al. 2017[36]** | Y | Y | CD | Y | Y | NA | Y | N | Y | NA | Y | NA | Y | N |
| **Norheim et al. 2017[37]** | Y | Y | Y | Y | NR | NA | Y | Y | Y | NA | Y | NA | N | Y |
| **Torres-Sánchez, Cabrera-Martos et al. 2017[38]** | Y | Y | Y | Y | Y | NA | Y | N | Y | NA | Y | NA | Y | CD |

Abbreviations: Y = Yes, N = No, NA = Not applicable, NR = Not reported, CD = Cannot determine
