# Supplementary material for: Change in skeletal muscle associated with unplanned hospital admissions in adult patients: A systematic review and meta-analysis
Source: PLoS One. 2019 Jan 4;14(1):e0210186. doi: 10.1371/journal.pone.0210186 (PMC6319740; doi:10.1371/journal.pone.0210186)
Supplement: S3 Table — (DOCX) [file pone.0210186.s004.docx]

**S3 Table. Description of usual care and interventions of included randomised control trials**

|  | **Description of Usual Care** | **Control group intervention on top of usual care** | **Treatment group intervention on top of usual care** |
| --- | --- | --- | --- |
| **Sloan et al. 1992[39]** | **Medical input:** Not described  **Therapy input:** Progress was reviewed daily by physiotherapist and occupational therapist. Standard rehabilitation consisted of progressive mobilization as tolerated with a walking aid. Upper extremity strengthening exercises were not performed.  **Nutritional input:** Not described. | **Intervention type:** Placebo injection.  **Intervention duration:** Weekly for duration of hospital stay, or for up to 4 weeks, whichever came first.  **Intervention dose:** 2mg/kg. | **Intervention type:** Nandrolone injections.  **Intervention duration:** Weekly for duration of hospital stay, or for up to 4 weeks, whichever came first.  **Intervention dose:** 2mg/kg. |
| **Saudny-Unterberger 1997[40]** | **Medical input:** Not described.  **Therapy input:** Not described.  **Nutritional input:** Subjects in both groups ordered their food and beverages from the hospital menu. | No additional treatment. | **Intervention type:** Oral nutritional supplements or extra snacks.  **Intervention duration:** 2 weeks.  **Intervention dose:** To ensure intake 1.5 times their resting energy expenditure (REE) if their BMI was normal (20 to 27) and at least 1.7 times REE if their BMI was below 20. |
| **Mets et al. 2004[41]** | **Medical input:** The physician in charge of treating patients’ medical conditions did not participate in the study. Acetaminophen could be given for fever or pain as necessary. Patients who required corticosteroids or NSAIDs in the course of hospitalisation were excluded from further follow-up.  **Therapy input:** All patients received standard physical therapy during the study period to maximize mobility and independence. This treatment was directed by physical therapists who were blinded to treatment-group assignment.  **Nutritional input:** Not described. | No additional treatment. | **Treatment group 1:**  **Intervention type:** Anti-inflammatory treatment: celecoxib.  **Intervention duration:** 2 weeks.  **Intervention dose:** 200mg, taken orally, once a day.  **Treatment group 2:**  **Intervention type:** Antipyetic treatment with acetanfinophen (paracetamol).  **Intervention duration:** 2 weeks.  **Intervention dose:** 1g orally, three times a day. |
| **Vermeeren et al. 2004[42]** | **Medical input:** Medical therapy (bronchodilator therapy and systemic glucocorticoids) was standardized, but slightly different between the two centres [further details of differences reported in paper]. Oxygen therapy was given if indicated based on resting arterial blood gas measurements.  **Therapy input: N**ot described.  **Nutritional input:** All the patients had the opportunity to select their own menus from a standardized hospital form. Dietetic consultation was standardized during the study period. | **Intervention type:** Vanilla flavoured water.  **Intervention duration:** 7 days.  **Intervention dose:** 3 times 125 ml with 0 MJ/day. | **Intervention type:** Oral nutritional supplement.  **Intervention duration:** 7 days.  **Intervention dose:** 3 times 125ml; 2.38MJ/day, 20 energy% protein, 20 energy% fat and 60 energy% carbohydrate. |
| **Troosters et al. 2010[43]** | **Medical input:** Patients received standard doses of oral corticosteroids to treat the exacerbation. In principle, patients received 32 mg·day oral methylprednisolone for 1 week, followed by 16 mg·day for 4 days and a subsequent decrease of 4 mg·week. However, steroids were given and tapered as judged possible by the treating chest physician.  **Therapy input:** Physiotherapy was limited to mucous secretion clearance techniques and breathing exercises. Patients were not restricted in their physical activities but no formal exercise therapy was offered.  **Nutritional input:** Not described. | No additional treatment. | **Intervention type:** Daily quadriceps resistance training (knee extension chair) supervised by physiotherapists.  **Intervention duration:** 7 days.  **Intervention dose:** The initial load was set at 70% of the 1 repetition maximum (the maximum load that can be moved only once over the full range of motion without compensatory movements). Patients performed three sets of eight repetitions and adjustments in the load were made based on symptoms. |
| **Beyer et al. 2011[44]** | **Medical input:** All patients, except those already using proton pump inhibitors, also received oral ranitidine 150 mg daily to avoid gastro-intestinal side effects. The medical staff was blinded to treatment allocation.  **Therapy input:** Not described.  **Nutritional input:** Not described. | **Intervention type:** Placebo.  **Intervention duration:** 10 days.  **Intervention dose:** 10 mg daily. | **Intervention type:** Piroxicam.  **Intervention duration:** 10 days.  **Intervention dose:** 10 mg daily. |
| **Borges et al. 2014[45]** | **Medial input:** Non-invasive ventilation if needed. Drug treatment and oxygen therapy were adjusted by the medical staff according to the Global Initiative for Chronic Obstructive Lung Disease recommendations.  **Therapy input:** Chest physiotherapy to remove bronchial secretions, and verbal instructions to carry on with their normative daily physical activities. Patients did not receive any exercise program or recommendation to exercise after hospital discharge.  **Nutritional input:** Not described. | No additional treatment. | **Intervention type:** Whole-body resistance training program for the upper (shoulder flexion and abduction, elbow flexion) and lower (knee extension and flexion, hip flexion) limbs by a physiotherapist in individualized sessions.  **Intervention duration:** Exercise training started on the third day of hospitalisation, and every patient completed a minimum of 3 sessions during the study.  **Intervention dose:** Exercise sessions were performed every morning with free weights in 2 sets of 8 repetitions in the sitting position.  The initial load was set at 80% of the load obtained in the 1-repetition maximum load test (maximum load exercise performed with dumbbells and anklets in the range of motion without any compensatory movements), and adjustments in the load during subsequent sessions were made based on symptoms. |
| **José et al. 2016[47]** | **Medical input:** Not described.  **Therapy input:** See control and intervention treatment descriptions.  **Nutritional input:** Not described. | **Intervention type: “**Standard respiratory physiotherapy”.  **Intervention duration:** 8 days.  **Intervention dose:** Daily 50 minute sessions. Involving secretion removal, breathing exercises and walking. Further details provided in paper). | **Intervention type:** “Physical training”  **Intervention duration:** 8 days  **Intervention dose:** Daily 50-minute sessions. Involving a warm-up, stretching, resistance exercises for peripheral muscles and aerobic walking training.  Initial load was 70% of maximum peripheral muscle strength. Adjustments made based on symptoms. Further details provided in paper. |
| **Martín Salvador et al. 2016[48]** | **Medical input:** Standard medical treatment according to medical prescription (in most cases oxygen therapy, antibiotics and bronchodilators).  **Therapy input:** Not described.  **Nutritional input:** Not described. | No additional treatment | **Intervention type:** “Standard treatment and a physiotherapy programme”.  **Intervention duration:** For duration of hospitalisation.  **Intervention dose:** 1 hour per day. Including “ventilatory re-education”, electrostimulation of both quadriceps (asymmetric biphasic current, pulse 400ms, 50 Hz frequency with cycles of 8 s contraction and 20 s of rest), and resistance exercises. The programme was the same for all patients and adapted to their levels of dyspnoea and fatigue. Further details provided in paper. |
| **Torres-Sánchez**  **2016[46]** | **Medical input:** Standard medical therapy including systemic steroids (76%), inhaled bronchodilators (100%) and oxygen.  **Therapy input:** Not described.  **Nutritional input: N**ot described. | No additional treatment. | **Intervention type:** “Multimodal pulmonary rehabilitation” provided by a physiotherapist.  **Intervention duration:** For duration of hospital stay, minimum of 7 sessions for patients included in analysis.  **Intervention dose:** Twice daily for 30­–45 minutes. Involving deep breathing exercises and limb exercises. Adjustments made based on symptoms. Further details published in paper. |
| **Torres-Sánchez, Valenza et al. 2017[49]** | **Medical input:** All patients received standard medical and pharmacological care (i.e., systemic steroids, inhaled bronchodilators, and oxy- gen), a regimen of oral prednisone or its equivalent in doses of 40–60 mg per day for the duration of therapy, taking into account the criteria of the doctor who follows each patient, which could change the medication according to the symptoms of the patients. The patients required antibiotic therapy during the exacerbation.  **Therapy input:** No supervised or progressive exercise was provided.  **Nutritional input:** not described. | No additional treatment. | **Intervention type:** Cycling exercise with a “pedal exerciser”.  **Intervention duration:** Second day of admission to discharge.  **Intervention dose:** Cycling time, velocity and intensity were adapted based on patients’ symptoms. Daily increment of time, velocity and intensity. Further details published in study. |
