# Supplementary material for: Change in skeletal muscle associated with unplanned hospital admissions in adult patients: A systematic review and meta-analysis
Source: PLoS One. 2019 Jan 4;14(1):e0210186. doi: 10.1371/journal.pone.0210186 (PMC6319740; doi:10.1371/journal.pone.0210186)
Supplement: S4 Table — Abbreviations: MAMC = Mid arm muscle circumference. (DOCX) [file pone.0210186.s005.docx]

**S6 Table: Sensitivity analysis examining effect of excluding studies that reported results as median and inter-quartile range**

| **Model** | **As described in manuscript** | **Excluding studies that reported median and interquartile range** |
| --- | --- | --- |
| Change in grip strength | Studies: n = 19  SMD = 0.10 (95% CI: 0.03: 0.16)  I^2^ = 78.66 | Studies: n = 15  SMD = 0.09 (95% CI: 0.02:0.16)  I^2^ = 78.90 |
| Change in knee strength | Studies: n = 15  SMD = -0.24 (95% CI: -0.33: -0.15)  I^2^ = 42.17 | Studies: n = 14  SMD = -0.23 (95% CI: -0.33 -0.13)  I^2^ = 34.97 |
| Change in MAMC | Studies: n = 7  SMD = -0.17 (95% CI: -0.22: -0.11)  I^2^ = 1.81% | Studies: n = 7  SMD = (-0.17 (95% CI: -0.23: -0.11)  I^2^ = 5.81% |

Abbreviations: MAMC = Mid arm muscle circumference
