# Supplementary material for: Change in skeletal muscle associated with unplanned hospital admissions in adult patients: A systematic review and meta-analysis
Source: PLoS One. 2019 Jan 4;14(1):e0210186. doi: 10.1371/journal.pone.0210186 (PMC6319740; doi:10.1371/journal.pone.0210186)
Supplement: S5 Table — (DOCX) [file pone.0210186.s006.docx]

**S3 Table. Data used in meta-analysis**

| **Change in grip strength** | |  |  |  |  |  |  |  |  |  |  |  |
| --- | --- | --- | --- | --- | --- | --- | --- | --- | --- | --- | --- | --- |
|  | **smd** | **2(1-r)** | **r** | **n** | **var_smd** | **se_smd** | **J** | **g** | **v_g** |  |  |  |
| Jones et al. 2017 | 0.348128808 | 0.403733068 | 0.798133466 | 67 | 0.006391015 | 0.079943825 | 0.994604317 | 0.346250415 | 0.006322233 |  |  |  |
| Torres-Sánchez, Cabera-Martos et al. 2017 | 0.037575758 | 0.77472824 | 0.61263588 | 52 | 0.014909138 | 0.122102981 | 0.992574257 | 0.03729673 | 0.014688537 |  |  |  |
| Matsuo et al. 2017 | 0.265072247 | 0.434684881 | 0.78265756 | 103 | 0.004368506 | 0.066094672 | 0.996305419 | 0.264092916 | 0.004336286 |  |  |  |
| Norheim et al. 2017 | 0.031473534 | 0.153655027 | 0.923172486 | 207 | 0.000742662 | 0.027251834 | 0.998175182 | 0.0314161 | 0.000739954 |  |  |  |
| Rossi et al. 2016 | -0.055217886 | 0.110579113 | 0.944710444 | 302 | 0.000366714 | 0.019149784 | 0.99875208 | -0.055148978 | 0.0003658 |  |  |  |
| Torres-Sánchez et al. 2016 | 0.003971119 | 0.195042871 | 0.902478565 | 25 | 0.007801776 | 0.088327665 | 0.984042553 | 0.00390775 | 0.00755477 |  |  |  |
| Martín Salvador et al. 2015 | -0.035260098 | 0.404885157 | 0.797557421 | 116 | 0.003492559 | 0.059097877 | 0.996724891 | -0.035144617 | 0.003469719 |  |  |  |
| Arezzo di Trifiletti et al. 2013 | 0.227272727 | 0.403719008 | 0.798140496 | 105 | 0.003944244 | 0.062803218 | 0.996376812 | 0.226449275 | 0.003915714 |  |  |  |
| Bodilsen et al. 2013 | -0.096256684 | 0.400102948 | 0.799948526 | 23 | 0.017476369 | 0.13219822 | 0.98255814 | -0.094577789 | 0.016872045 |  |  |  |
| Wieboldt et al. 2012 | 0.085889571 | 0.401219466 | 0.799390267 | 13 | 0.030976874 | 0.176002484 | 0.967391304 | 0.083088824 | 0.028989582 |  |  |  |
| Humphreys et al. 2002 | 0.222222222 | 0.26408179 | 0.867959105 | 50 | 0.005412047 | 0.073566613 | 0.989726027 | 0.219939117 | 0.005301411 |  |  |  |
| Karlsen et al. 2017 | 0.007627765 | 0.163435485 | 0.918282258 | 88 | 0.001857275 | 0.043096119 | 0.99566474 | 0.007594697 | 0.001841207 |  |  |  |
| Beyer et al. 2011 | 0.302691542 | 0.962825498 | 0.518587251 | 14 | 0.071923827 | 0.26818618 | 0.971153846 | 0.293960055 | 0.067834223 |  |  |  |
| Bautmans et al. 2005 | 0.1 | 0.4 | 0.8 | 63 | 0.006380952 | 0.079880864 | 0.993902439 | 0.099390244 | 0.006303373 |  |  |  |
| Mets et al. 2004 | 0.271582181 | 0.4 | 0.8 | 15 | 0.027650092 | 0.166283167 | 0.972222222 | 0.264038232 | 0.02613531 |  |  |  |
| Vermeeren et al. 2004 | 0 | 0.111111111 | 0.944444444 | 22 | 0.005050505 | 0.071066905 | 0.98255814 | 0 | 0.004875861 |  |  |  |
| Gupta et al. 2001 | 0.042461342 | 0.407605815 | 0.796197093 | 17 | 0.023998427 | 0.154914258 | 0.975806452 | 0.041434052 | 0.02285126 |  |  |  |
| Saudny Unterberger et al. 1997 | 0.042517007 | 0.095104406 | 0.952447797 | 10 | 0.009519037 | 0.09756555 | 0.955882353 | 0.040641257 | 0.008697649 |  |  |  |
| Potter et al. 1995 | 0.422693267 | 0.928988315 | 0.535505843 | 41 | 0.024682422 | 0.157106403 | 0.992424242 | 0.419491045 | 0.024309862 |  |  |  |
| Sloan et al. 1992 | 0.090909091 | 0.4 | 0.8 | 14 | 0.028689492 | 0.169379728 | 0.97 | 0.088181818 | 0.026993943 |  |  |  |
|  |  |  |  |  |  |  |  |  |  |  |  |  |
| **Change in knee extension strenght** | | |  |  |  |  |  |  |  |  |  |  |
| **study** | smd | 2(1-r) | r | n | var_smd | se_smd | J | g | v_g |  |  |  |
| Torres-Sánchez, Valenza et al. 2017 | -0.565095921 | 1.588419936 | 0.205790032 | 29 | 0.063518542 | 0.252028851 | 0.986363636 | -0.557390068 | 0.061798029 |  |  |  |
| Torres-Sánchez, Cabera-Martos et al. 2017 | -0.640625 | 0.987539063 | 0.506230469 | 52 | 0.022888121 | 0.151288204 | 0.984693878 | -0.630819515 | 0.022192826 |  |  |  |
| Torres-Sánchez et al. 2016 | -0.413333333 | 0.345744 | 0.827128 | 25 | 0.015011129 | 0.122519912 | 0.984042553 | -0.406737589 | 0.014535873 |  |  |  |
| Martín-Salvador et al. 2016 | -0.361846902 | 0.392487787 | 0.803756107 | 20 | 0.020909131 | 0.1445999 | 0.97972973 | -0.354512167 | 0.020070055 |  |  |  |
| Martín Salvador et al. 2015 | -0.27134167 | 0.834192661 | 0.582903669 | 116 | 0.007456051 | 0.086348428 | 0.996724891 | -0.270452996 | 0.007407292 |  |  |  |
| Borges et al. 2014 | -0.26718886 | 0.154068206 | 0.922965897 | 14 | 0.01139769 | 0.106759965 | 0.97 | -0.259173194 | 0.010724087 |  |  |  |
| Mesquita et al. 2013 | -0.090277778 | 0.803858025 | 0.598070988 | 20 | 0.040356689 | 0.200889743 | 0.979166667 | -0.088396991 | 0.038692676 |  |  |  |
| Bodilsen et al. 2013 | 0.226851852 | 0.874849966 | 0.562575017 | 21 | 0.042731458 | 0.206715888 | 0.984375 | 0.223307292 | 0.041406533 |  |  |  |
| Wieboldt et al. 2012 | 0.266666667 | 0.824320842 | 0.587839579 | 13 | 0.065663848 | 0.256249582 | 0.967391304 | 0.257971014 | 0.061451245 |  |  |  |
| José et al. 2016 | -0.213333333 | 0.537777778 | 0.731111111 | 17 | 0.032353836 | 0.179871721 | 0.975806452 | -0.208172043 | 0.030807265 |  |  |  |
| Burtin et al. 2013 | -0.073910297 | 0.827273435 | 0.586363283 | 19 | 0.043659633 | 0.208948876 | 0.97 | -0.071692988 | 0.041079348 |  |  |  |
| Crul et al. 2010 | -0.2 | 0.101239669 | 0.949380165 | 9 | 0.011473829 | 0.107115961 | 0.95 | -0.19 | 0.010355131 |  |  |  |
| Crul et al. 2007 | -0.138461538 | 0.104378698 | 0.947810651 | 14 | 0.007527089 | 0.0867588 | 0.97 | -0.134307692 | 0.007082238 |  |  |  |
| Pitta et al. 2006 | -0.333333333 | 0.284444444 | 0.857777778 | 17 | 0.017661583 | 0.132896889 | 0.975806452 | -0.325268817 | 0.016817328 |  |  |  |
| Spruit et al. 2003 | -0.227272727 | 2.905475207 | -0.452737603 | 25 | 0.124188054 | 0.352403255 | 0.984375 | -0.223721591 | 0.120337497 |  |  |  |
|  |  |  |  |  |  |  |  |  |  |  |  |  |
| **Change in mid-arm muscle circumference** | | |  |  |  |  |  |  |  |  |  |  |
| Study | smd | 2(1-r) | r | n | var_smd | se_smd | J | g | v_g |  |  |  |
| Sloan et al. 1992 | 0.070588235 | 2.004429066 | -0.002214533 | 14 | 0.1435302 | 0.378853798 | 0.97 | 0.068470588 | 0.031852385 |  |  |  |
| Potter et al. 1995 | -0.19236381 | 0.08325863 | 0.958370685 | 41 | 0.00206827 | 0.045478238 | 0.992990654 | -0.191015466 | 0.002039377 |  |  |  |
| Unosson et al. 1995 | -0.162424233 | 2 | -2.22045E-16 | 50 | 0.040527633 | 0.20131476 | 0.992268041 | -0.161168375 | 0.009276272 |  |  |  |
| Gupta 2001 | -0.129694323 | 2.000038138 | -1.9069E-05 | 17 | 0.118638769 | 0.34443979 | 0.975806452 | -0.126556557 | 0.026264727 |  |  |  |
| Weinsier et al. 1979 | -0.409431668 | 2 | -2.22045E-16 | 29 | 0.07474601 | 0.273397165 | 0.986363636 | -0.403848509 | 0.016905434 |  |  |  |
| Abad et al. 1986 | -0.090057593 | 0.327640144 | 0.836179928 | 47 | 0.006999336 | 0.083662034 | 0.991758242 | -0.373156832 | 0.048070775 |  |  |  |
| Antonelli Incalzi et al. 1996 | -0.128205128 | 2 | 2.22045E-16 | 283 | 0.007125218 | 0.084411003 | 0.99871134 | -0.128039915 | 0.001652123 |  |  |  |
|  |  |  |  |  |  |  |  |  |  |  |  |  |
| **Exercise intervention** | |  |  |  |  |  |  |  |  |  |  |  |
| Study | cg0_n | cg0_m | cg0_s | ig0_n | ig0_m | ig0_s | cg1_n | cg1_m | cg1_s | ig1_n | ig1_m | ig1_s |
| Borges et al. 2014 | 14 | 292.24 | 114.74 |  | 257.91 | 93.16 | 14 | 263.01 | 42.04 | 15 | 268.23 | 56.43 |
| Jose et a. 2016 | 17 | 237.32 | 81.40 | 32 | 251.05 | 55.90 | 17 | 221.63 | 65.70 | 32 | 318.72 | 83.36 |
| Torres-Sánchez 2016 | 25 | 153.50 | 75.00 | 24 | 126.90 | 36.20 | 25 | 122.50 | 42.04 | 24 | 143.40 | 56.43 |
| Torres-Sánchez, Valenza et al. 2017 | 29 | 101.50 | 13.52 | 29 | 103.36 | 11.36 | 29 | 86.33 | 40.17 | 29 | 114.03 | 37.31 |
| Martín-Salvador 2016 | 20 | 100.39 | 52.04 | 24 | 105.76 | 33.74 | 20 | 86.71 | 29.40 | 24 | 113.98 | 44.68 |
